# An Example of How Barcodes Can Clarify Cryptic Species: The Case of the Calanoid Copepod *Mastigodiaptomus albuquerquensis* (Herrick)

**DOI:** 10.1371/journal.pone.0085019

**Published:** 2014-01-21

**Authors:** Martha Angélica Gutiérrez-Aguirre, Adrián Cervantes-Martínez, Manuel Elías-Gutiérrez

**Affiliations:** 1 Universidad de Quintana Roo, Cozumel Quintana, Roo, Mexico; 2 El Colegio de la Frontera Sur, Chetumal Quintana, Roo, Mexico; Auburn University, United States of America

## Abstract

**Background:**

The freshwater calanoid *Mastigodiaptomus* is a genus with high richness in the Americas and is composed of nine species, seven recorded in Mexico and four that are apparently endemic to small areas. *Mastigodiaptomus albuquerquensis* is a common, widely distributed species ranging from the southern USA to Central America. This species can be easily identified by a notable butterfly-like sclerotization on the basis of the right fifth leg of males. Nevertheless, morphological differences observed among populations throughout this species distributional range have led to the description of several related species or subspecies, such as *M. albuquerquensis patzcuarensis* from Lake *Pátzcuaro* in the Central Plateau of Mexico.

**Methods:**

Genetic results based on barcodes, morphology based on scanning electron and light microscopy images, and morphometric analyses were used to describe cryptic species within the *M. albuquerquensis* complex.

**Results:**

The morphological analyses coincided partially with the genetic markers, suggesting the existence of at least two sibling species: *M. albuquerquensis* s. str. and *M. patzcuarensis*. A third species was genetically separated but was morphologically indistinguishable from the *M. patzcuarensis* group.

**Conclusions:**

Hidden diversity has been a major problem in establishing real patterns of species distribution and genetic acquisition from megadiverse hotspots such as Mexico, where the Nearctic and the Neotropical regions of the Americas meet. Barcodes can help taxonomists to reveal and formally name these new species. Here, we describe two of three potential species highlighted by the use of barcodes: *M. albuquerquensis* s. str. in the northern semi-desert and *M. patzcuarensis* on the Central Plateau at more than 2000 m above sea level.

## Introduction

*Mastigodiaptomus* is a freshwater calanoid genus with nine species in the Americas, four of them (*M. montezumae*, *M. reidae*, *M. maya*, and *M. suarezmoralesi*) are apparently endemic to Mexico, one is found in Guatemala (*M. amatitlanensis*) and one (*M. purpureus*) is from Cuba [1], [2]. In contrast, *Mastigodiaptomus albuquerquensis* (Herrick, 1895) is a common and widely distributed species, ranging from the southern USA to Central America [1]. In Mexico, this species has been recorded from the Baja California Peninsula to the Yucatan Peninsula [1], [3], [4].

*Mastigodiaptomus albuquerquensis* s. l. is easily identified by a butterfly-like sclerotization on the basis of the right fifth leg of males. In the last 50 years, every specimen with this type of sclerotization has been assigned to this species. Barcoding has confirmed earlier suspicions of overlooked diversity [5]. The morphological differences observed, such as notable changes in body size, were previously explained by the life history hypothesis, supporting the idea that morphological variations are the result of natural selection in response to particular ecological problems faced by species with wide distributional ranges [6].

Nevertheless, on the basis of subtle morphological characters, the population in Pátzcuaro Lake was assigned to a different subspecies, *M. albuquerquensis patzcuarensis* [7]. Previously *Diaptomus lehmeri* was described from Mexico City [8], although this species was later synonymized with *M. albuquerquensis* [9].

However, the genetic analysis of Copepoda demonstrated that under the name “*M. albuquerquensis*” is present a complex of at least two species in Mexico [5]. Similarly, after one initial study, ecophysiological analyses, hybridization assays, and fine morphological details together with barcoding demonstrated the validity of another species, *Leptodiaptomus garciai* Osorio Tafall, 1942, which is micro-endemic to a saline crater lake located at 2365 masl [10].

The goal of the present work was to analyze in detail the morphology of the groups highlighted by the barcodes within *Mastigodiaptomus albuquerquensis* to ascertain the true status of this species complex and to define the taxonomic status of the surveyed populations.

## Materials and Methods

### Ethics statement

We collected from several freshwater ecosystems in Mexico. Zooplankton is not under any protection by Mexican laws; thus, no specific permits for this type of field studies are needed.

### Sampling

Biological samples were obtained using a plankton net with a 50 µm mesh; the material was fixed and preserved in 100% ethanol. Samples were collected along a latitudinal gradient between 17 and 31°N, which is within the known distributional range of *M. albuquerquensis*.

The type locality of *M. albuquerquensis* (“water reservoir supplying Albuquerque, New Mexico”) [11] appears no longer to exist, but all the original samples were taken around the Rio Grande Basin (named the Río Bravo in Mexico, which constitutes the physical border between Mexico and the USA). After several surveys in the region, the closest place to the type locality for this species was ∼200 km away and is part of the same biogeographic province; thus, we concluded that this material represents *M. albuquerquensis* s. str. Adults males and females taken here were considered as topotypes for the morphological and molecular analyses. The material examined and accession numbers of the sequences and coordinates are summarized in Table S1.

### Morphological observations

The specimen analysis was performed following the current standards for the taxonomic study of diaptomid copepods [12]. Structures of each specimen considered to have taxonomic value were dissected, and appendages were mounted in glycerin. Taxonomically important structures were illustrated with the aid of a camera lucida. Some specimens were prepared for SEM (scanning electron microscopy) for the observation of microcharacters. Abbreviations in the descriptive section are as follows: P1–P4, first to fourth swimming legs; Exp, exopod; Enp, endopod; Bsp, basis; s, seta(e); ae, aesthetasc; sp, spine; sps, spiniform process; Fu, furcal rami.

To complement the morphological analysis, the type specimens of *M. albuquerquensis patzcuarensis* were examined (slides 04057, 04058, 04059, and 04060) as well as specimens of *M. albuquerquensis* from Arizona, USA (slides 02965, 02966, 02967, and 02968); all of them deposited in the Staatliches Museum für Naturkunde, Karlsruhe, Germany.

Finally, meristic magnitudes transformed as the square root of a + ā of adult females and males from the different populations were examined with a principal component analysis (PCA) performed with the Multi Variate Statistical Package (MVSP 3.21).

In females, 20 morphological features were considered in the PCA: total body length, prosomal length, first prosomite length, A1 length (posterior or anterior to the furcal rami), urosomal length without Fu, urosomal length with Fu, Fu length, length (L)-to-width (W) ratio of the rostral spines, genital double-somite L, genital double-somite W, L/W ratio of genital double-somite, point of insertion of the left spine on the genital double-somite (%), point of insertion of the right spine on the genital double-somite (%), L/W ratio of the left spine on the genital double-somite, L/W ratio of the right spine on the genital double-somite, distance between spines on the right prosomite wings, distance between spines on the left prosomite wings, L ratio of ExpP5/EnpP5, L ratio of EnpP5/distal setae of EnpP5, and the number of eggs carried in ovisacs. A total of 94 adult females from Pátzcuaro Lake, Cuitzeo, Hueco Tanks, Rancho Grande, Laguna Bustillos, Arizona, La Cruz, El Salvador, La Goleta dam, Ignacio Ramírez dam, and km 55 pond were analyzed in the PCA.

For the males, 12 morphological features were considered in the PCA: total body length, prosomal length, first prosomite length, urosomal length without Fu, urosomal length with Fu, Fu length, L/W ratio of the rostral spines, L/W ratio of the 20^th^ segment of A1, L ratio of the 21^st^/20^th^ segments of A1, L ratio of spines on the 10^th^/11^th^ segments of A1, L ratio of 20^th^/21^st^ segments of A1, L/W ratio of the rostral spines, and L/W ratio of the right spine on the first urosomite. A total of 69 adult males from Pátzcuaro Lake, Cuitzeo, Laguna La Cruz, El Salvador, Hueco Tanks, Rancho Grande, Laguna Bustillos, Arizona, La Goleta dam, Ignacio Ramírez dam, and km 55 pond were analyzed in the PCA.

### Molecular markers

A total of 53 specimens were sequenced. Genomic DNA was extracted using a membrane-based approach employing AcroPrep 96, 1 ml filter plates with 3.0 µm glass fiber media over a 0.2 µm BioInert membrane (PALL) [13], or a modified HotShot technique [14]. A 658 bp segment of COI was amplified using LCOI490 and HCO2198 primers [15] or the new Zplank primers [16]. The 12.5 µl PCR reaction mixes included 6.25 µl of 10% trehalose, 2 µl of ultrapure water, 1.25 µl of 10X PCR buffer, 0.625 of MgCl_2_ (50 mM), 0.125 µl of each primer (0.01 mM), 0.0625 µl of each dNTP (0.05 mM), 0.625 µl of Taq polymerase (New England Biolabs or Invitrogen), and 2.0 µl of DNA template. PCR products were visualized on pre-cast agarose gels (E–Gels©, Invitrogen), and the most intense products were selected for sequencing. Sequence analysis was carried out at the Canadian Centre for DNA Barcoding using standard protocols [17].

Products were labeled with a BigDye© Terminator v. 3.1 Cycle Sequencing Kit (Applied Biosystems, Inc.) and sequenced bidirectionally on an ABI 3730 capillary sequencer.

### Sequence alignment and analysis

Bidirectional sequences were edited using CodonCode v. 3.0.1 (CodonCode Corporation, Dedham, Massachusetts) and uploaded to the Barcode of Life Database (BOLD; http://www.barcodinglife.org). Sequence data, trace files, and primer details for all specimens are available within the BOLD project files *Mastigodiaptomus* of Mexico (MALB), Zooplankton of Mexico and Central America (ZPLMC), Zooplankton II (ZPII), and Zooplankton III (ZMIII). Accession numbers for BOLD and GenBank are listed in Table S1.

We used the tools provided by BOLD to obtain the Id tree: Sequence divergences were calculated using the Kimura two-parameter (K2P) distance model [18]. Neighbor-joining (NJ) trees of K2P distances were created to provide a graphic representation of the patterning of divergence between species (Figure S1). Additionally, to compare these results in Mega 5 [19], sequences with at least 604 bp (except two from Rancho Grande to avoid a singleton, see Figure S1) were aligned with CLUSTAL W using default parameters, and phylogenetic relationships of all haplotypes were then inferred using maximum likelihood analysis. A discrete Gamma distribution was used to model evolutionary rate differences among the sites (five categories (+G, parameter = 0.7043)). All positions with less than 0% site coverage were eliminated. In all cases, a sequence of *Mastigodiaptomus* cf. *nesus* was used as the outgroup.

### Nomenclatural acts

The electronic edition of this article conforms to the requirements of the amended International Code of Zoological Nomenclature, and hence the new names contained herein are available under that Code from the electronic edition of this article. This published work and the nomenclatural acts it contains have been registered in ZooBank, the online registration system for the ICZN. The ZooBank LSIDs (Life Science Identifiers) can be resolved and the associated information viewed through any standard web browser by appending the LSID to the prefix “http://zoobank.org/”. The LSID for this publication is: urn:lsid:zoobank.org:pub:FF036BBC-2CB6-4C1D-932F-9C8FB8377C82. The electronic edition of this work was published in a journal with an ISSN, and has been archived and is available from the following digital repositories: PubMed Central, LOCKSS.

## Results

### Molecular analyses

**Figure 1:**
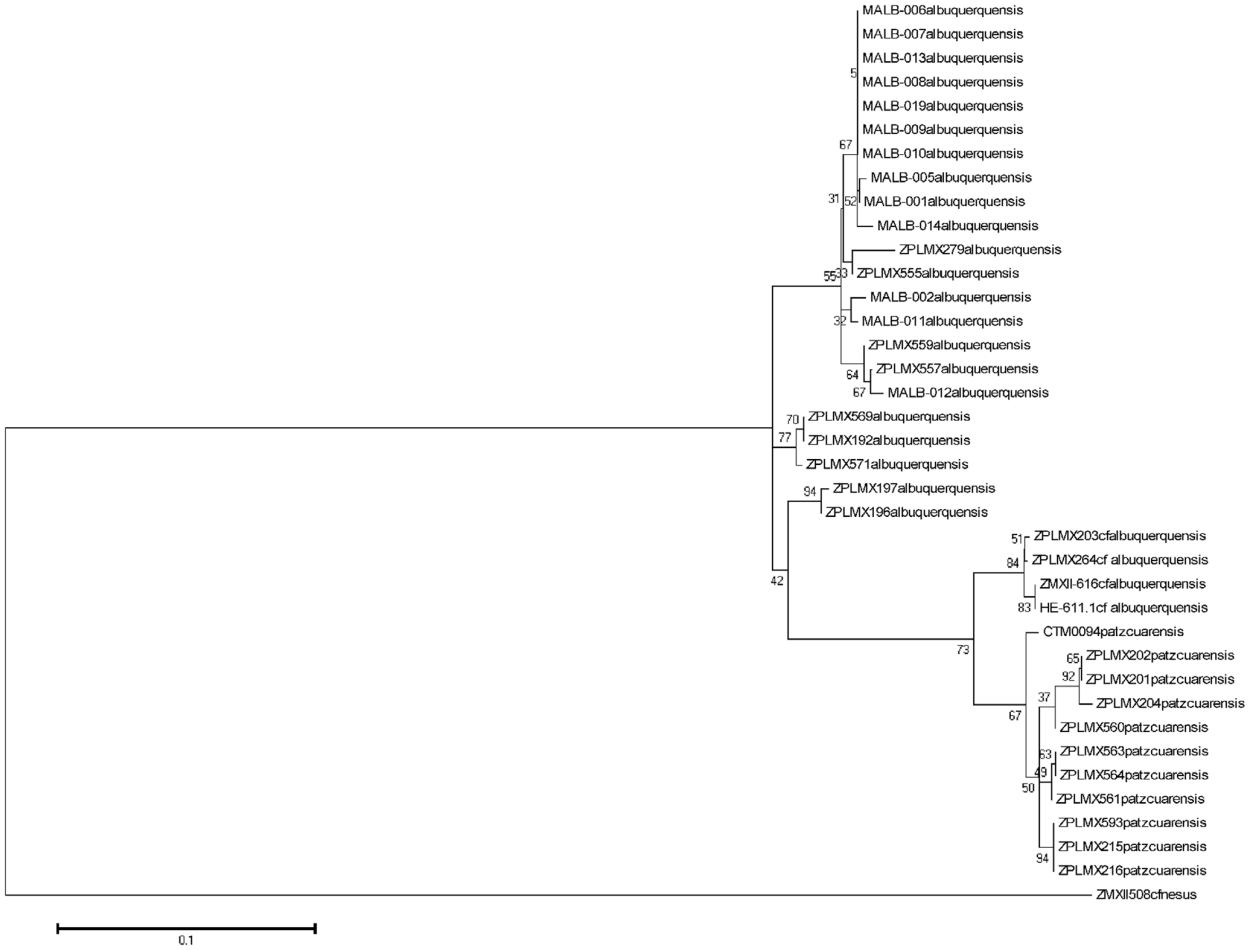
Id tree inferred by using the maximum likelihood method as described in the Methods section.

DNA barcoding, barcode index numbers (BIN), and 2% of MOTU provided by BOLD suggest the presence of five possible species based on the COI haplotypes (Figure S1, Fig. 1). The results of the K2P analyses in BOLD and the maximum likelihood method were identical. The latter analysis found only one tree (Fig. 1) on which bootstrap percentages are shown. Average values are shown in Table 1.

**Table 1:** Genetic divergences (K2P) at different taxonomic levels for copepods.

	n	Taxa	Comparisons	Min Dist (%)	Mean Dist (%)	Max Dist (%)	SE Dist (%)
Within species	53	3	542	0	1.89	6.54*	0.003
Within genus	53	1	836	1.44	8.38	50	0.004

* Value reflects the divergence between probable cryptic species of *M. albuquerquensis*.

The first group belongs to the species *M. albuquerquensis*, found in the northern semi-desert region, and is recognized as the strict form. This group forms a consistent cluster, and all localities for this species are the closest ones to the no longer extant type locality. The second main species is apparently restricted to the Central Plateau in localities close to Lago de Pátzcuaro. This taxon, described as *M. albuquerquensis patzcuarensis* Kiefer, 1938 [7], is in fact a full species, *Mastigodiaptomus patzcuarensis*, and also forms a consistent cluster.

A third group suggested by the genetic analyses seems to be sympatric with *M. patzcuarensis* in systems such as Cuitzeo, and which appears in several other water bodies in the Central Plateau, is here regarded as *M.* cf. *albuquerquensis*. Two other groups remaining as possible different species are the specimens from Rancho Grande to Zacatecas, with an average divergence of 4.4% from the main cluster, and two specimens from Papasquiaro B, with an average divergence of 4.6%. As these sequences are shorter than the others and present low- to middle-quality trace files, they are here regarded as members of *M. albuquerquensis* s. str. on the basis of their morphology and geographic location in the northern semi-desert region.

### PCA analysis

**Figure 2:**
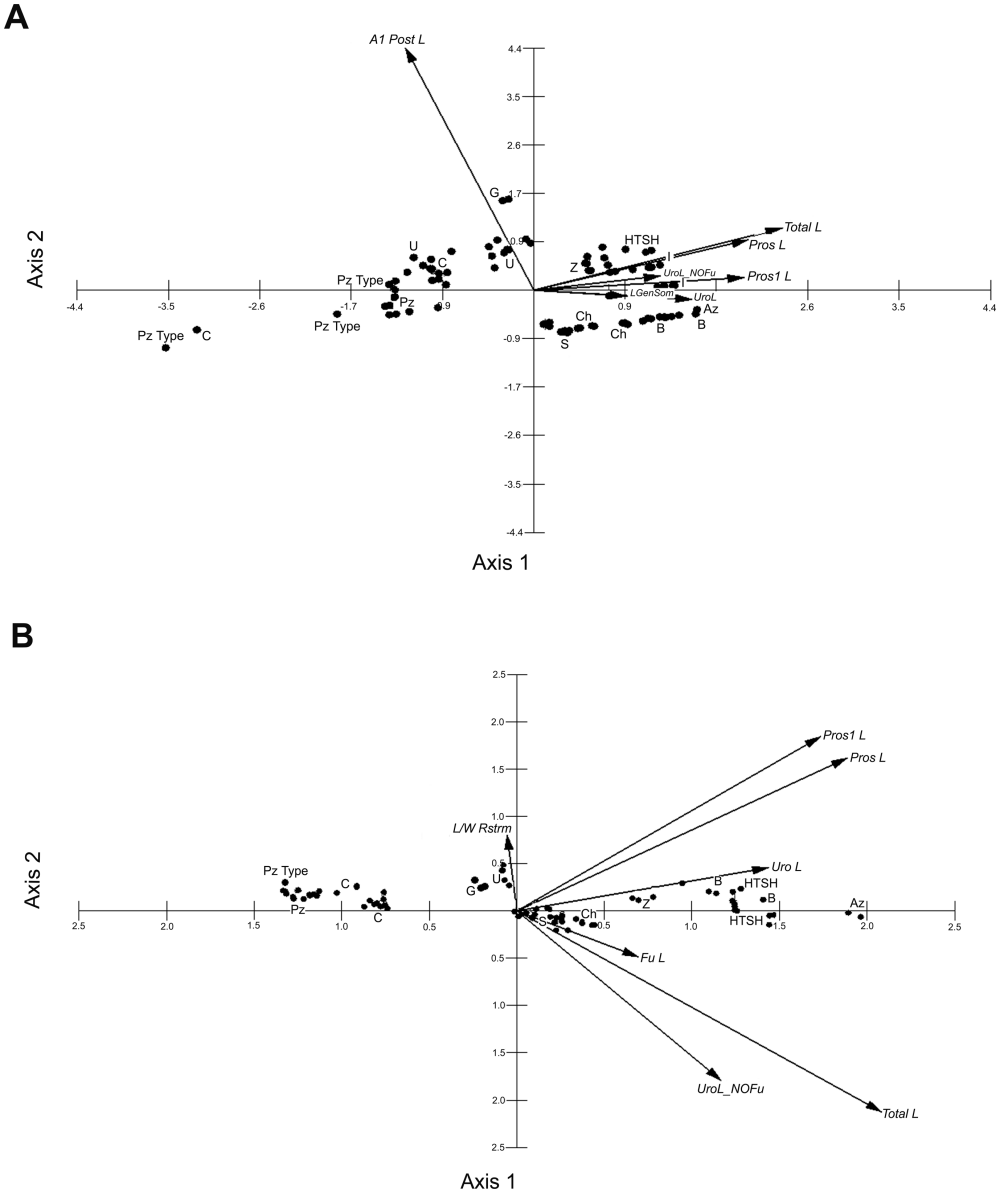
PCA biplot of the females (A) and males (B) analyzed. Cuitzeo = C; Pátzcuaro = Pz; Goleta = G; La Cruz = U; Ignacio Ramírez = I; Hueco Tanks = HTSH; Rancho Grande, Zacatecas = Z; Arizona = AZ; km 55 pond = Ch; El Salvador = S; and Bustillos = B. The type specimens of *Mastigodiaptomus albuquerquensis patzcuarensis* are labeled as PzType. A1 Post L = A1 length (posterior or anterior to the furcal rami); Pros L = prosomal length; Total L = total body length; UroL_NOFu = urosomal length without Fu; L GenSom = genital double-somite length; Pros1 L = first prosomite length; UroL = urosomal length with Fu; Fu L = furcal rami length; and L/W Rstrm = ratio of rostral spines.

From the biplot, a gradient was observed from axis 1 to axis 2, representing the meristic features in the specimens. Axis 1+2 explained 82.6% of the variability in females (Fig. 2A) and 91.8% in males (Fig. 2B). Two defined groups were found in each case: one group included the surveyed specimens from Pátzcuaro, Cuitzeo, La Cruz, and Goleta. The second group included the available specimens from Hueco Tanks, Arizona, Rancho Grande (Zacatecas), Ignacio Ramírez, El Salvador, km 55 pond, and Bustillos (Fig. 2A, B).

### Morphological analyses

The morphology of A1, the mouthparts, and thoracic legs in females and males, as well as the fifth leg from the males, are similar along all latitudinal gradients studied. Morphological similarities are described in this section; the detailed morphological differences between the genetically different materials will be described later in this paper.

#### Female antennules (and left A1 in males)

25-segmented, each segment armed with setae, spines, or aesthetasc in the following order: (1)2 ae; (2)3s+1ae; (3)1s+1ae; (4)1s; (5)2 ae; (6)1s; (7)1s+1ae; (8)1ae+1 sp; (9)1s+2 ae; (10)1s; (11)1s+1ae; (12)2ae+1sp; (13)1s; (14)1s+1ae; (15)1s; (16)1s+1ae; (17)1s; (18)1s; (19)1s+1ae; (20)1s; (21)1s; (22)2s; (23)2s; (24)2s; (25)5s.

#### Right A1 males

**Figure 3:**
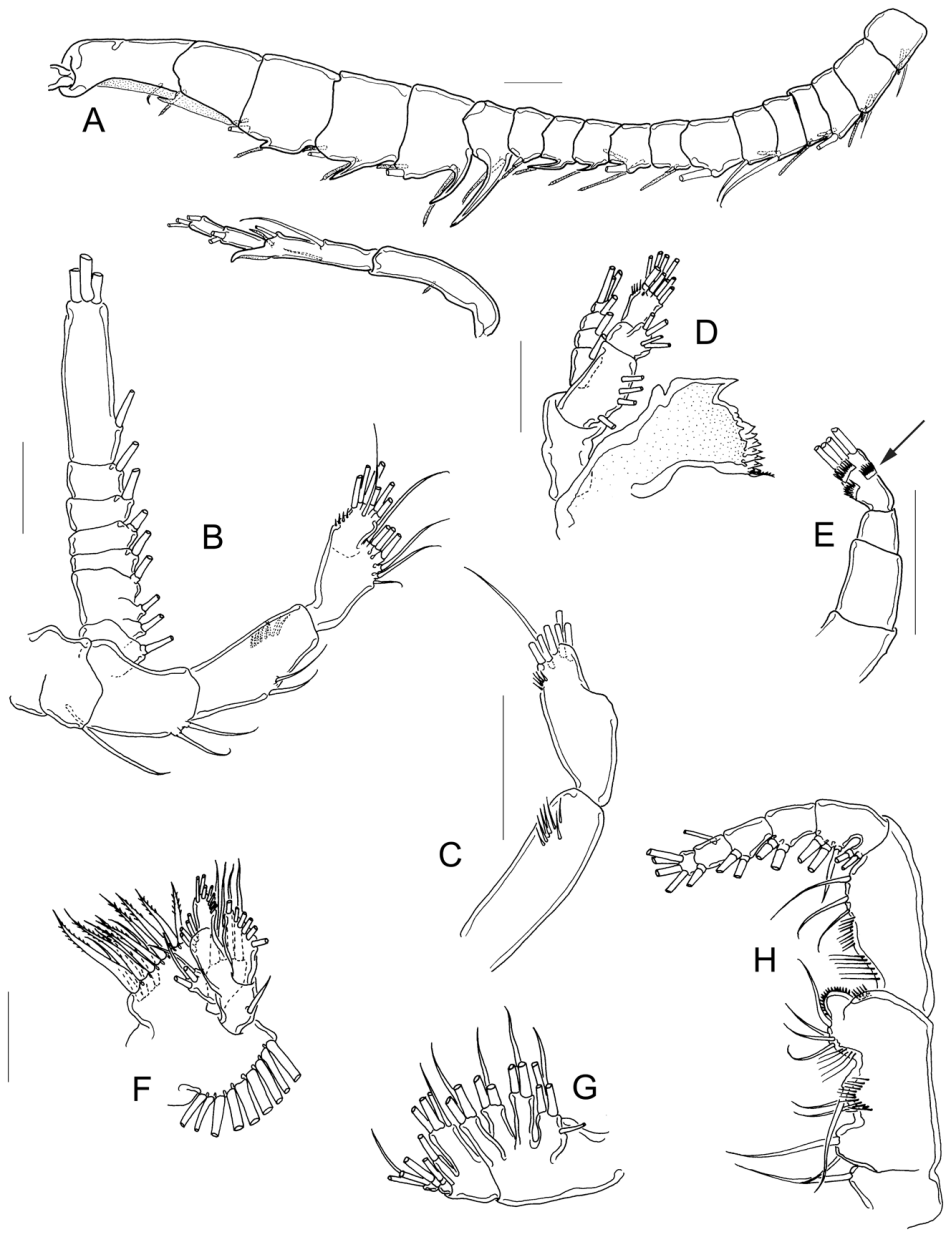
Morphology of the cephalic appendages. A, A1; B, A2; C, EnpA2; D, mandible; E, detail of Enp2 of mandible; F, maxillule; G, maxilla; H, maxilliped. A: male from Rancho Grande, Zacatecas; C, E: typus of *M. albuquerquensis patzcuarensis*, slide 4057; B, D, F–H: female from Rancho Grande, Zacatecas.

22-segmented, each segment armed with setae, spines, spiniform process, or aesthetasc in the following order: (1)1s+1ae; (2)1s+2ae; (3)1s+1ae; (4)1ae; (5)1s+1ae; (6)1s; (7)1s; (8)1ae+1sp; (9)1s+2ae; (10)1ae+1sps, (11)1ae+1sps; (12)1ae+1sp; (13)1ae+1sps; (14)1s +2ae+1sps; (15)1s +2ae+1sps; (16)1s+ 2ae+1sps; (17)1ae; (18)1ae; (19)1ae; (20)3s+1ae; (21)2s; (22)3s. (Fig. 3A).

#### Antennae

Exp longer than Enp (Fig. 3B). Coxa with one long seta; long Bsp bearing two setae. Enp bi-segmented; Enp1 with two medial setae plus a group of long spine-like setae (Fig. 3B, C); Enp2 bilobed, inner lobe with a row of spine-like setae and seven long setae. Outer lobe of Enp2 with seven setae. Exp 7-segmented; setation pattern on each segment as follows: 1, 3, 1, 1, 1, 1, 4.

#### Mandible

strong, toothed gnathobase with a movable seta at the tip (Fig. 3D). Coxa long, nude; Bsp with four setae. Enp bi-segmented; Enp1 with four setae; Enp2 quadrangular, with nine distal long setae (Fig. 3D) plus three toothed pectens on outer margin (arrowed in Fig. 3E). Exp four-segmented, with a 1, 1, 1, 3 setation pattern.

#### Maxillule

praecoxal arthrite with 12 spiniform setae, eight anterior, four posterior (Fig. 3F). Coxal epipodite with nine long setae. Coxal endite with four long setae. Bsp with one internal lobe bearing four setae; basal exite with one seta. Basal endite with four setae. Enp with one segment bearing seven setae. Exp plate-like, with six long setae.

#### Maxilla

indistinctly segmented; first praecoxal lobe with five setae, second praecoxal lobe with three setae (Fig. 3G). Two coxal lobes with three setae each. Two well-developed basal lobes: proximal lobe bearing four setae, distal lobe with one seta. Enp two-segmented; Enp1 bearing two setae, Enp 2 with three setae.

#### Maxilliped

praecoxa with one long seta; coxa with three distinct lobes bearing nine setae (2, 3, 4), plus two rows of spine-like setae, and protuberance bearing short spinules surrounding the process (Fig. 3H). Bsp with a group of three distal setae and two rows of spine-like setae. Six-segmented Enp; Enp1 partially fused to Enp2. Setation pattern as follows: 2, 3, 2, 2, 2, 4.

#### Swimming legs

P1 with three-segmented Exp and two-segmented End; P2–P4 with three-segmented Exps and Enps. The armature formula of P1–P4 observed in the material analyzed here and shown in Table 2 has been described and illustrated for other *Mastigodiaptomus* species [1], [20]. Only two females from Zacatecas (12.5% of the surveyed population) showed two spines on Enp3P4 (on the left side).

**Table 2:** Setation formula for major armament of swimming legs (P1–P4) in the females and males in all the species described here.

	Coxa	Basis	Exp	Enp
P1	0-1	0-0	I-1; 0-1; I-3-2	0-1; 1-2-3
P2	0-1	0-0	I-1: I-1; I-3-3	0-1; 0-2; 2-2-3
P3	0-1	0-0	I-1; I-1; I-3-3	0-1; 0-2; 2-2-3
P4	0-1	0-0	I-1; I-1; I-3-3	0-1; 0-2; 2-2-3

Roman numerals indicate spines, and Arabic numbers are setae.

#### P5, males (posterior view) (Fig. 4A)

**Figure 4:**
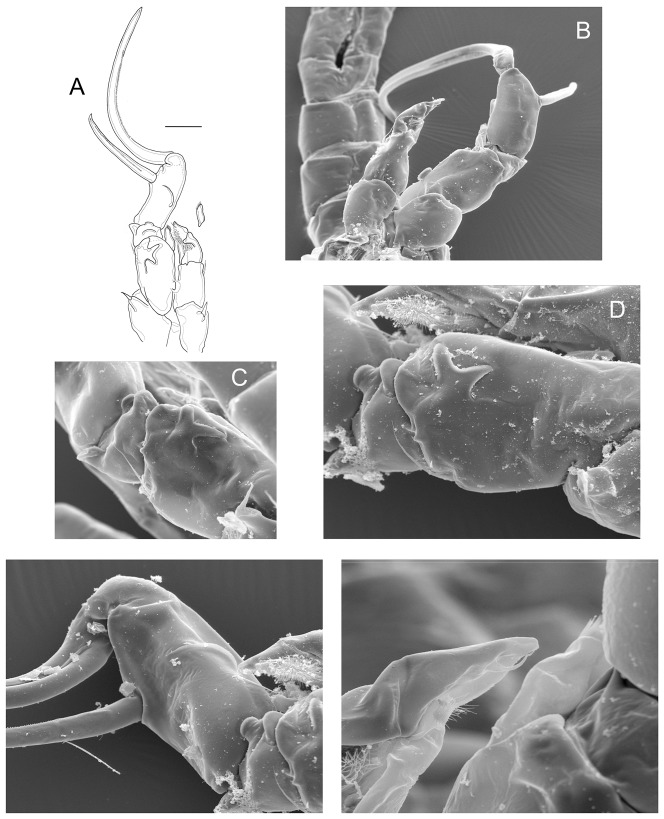
Morphology of the fifth leg in males. A, P5 posterior view; B, P5 anterior view; C, D P5 detail of right basis posterior view; E, P5 detail of right Exp2 posterior view; F, P5 detail of left Exp2. A–C: males from Cuitzeo; D–F: males from El Salvador, Durango.

coxal segments with strong spines. Right Bsp with a proximal bulb; one rounded, hyaline membrane medially (arrowed in Fig. 4B) and one distal, butterfly-like sclerotization (Fig. 4C, D). Right Enp as long as Exp1; Exp2 bearing one curved, medial hyaline membrane (arrowed in Fig. 4E); aculeus inserted at distal half of the segment (Fig. 4A, E). Aculeus armed with spines on the medial edge and with a curved, sharp tip; terminal claw very long, armed, bent, with a dagger-like tip (Fig. 4A). Left Bsp bearing one lateral seta, one-segmented Enp, and two-segmented, pilose Exp. Exp2 with one fine, tiny seta (Fig. 4A, F).

From the PCA analyses, the variability is explained mainly by features related to the body length and secondarily by the number of eggs carried by females in axis 1, whereas the length of the A1 related with the Fu is more important in axis 2 (Fig. 2A).

In males, magnitudes related to the body length are important to explaining the variability in axis 1, whereas the L/W ratio of rostral spines is important in axis 2 (Fig. 2B).

In contrast to the genetic analyses, the PCA suggested the presence of only two sibling species with disjunct distributions in Mexico (Fig. 2), both coincident with two of the three main groups according to the barcodes, namely, *M. albuquerquensis* s. str. and *M. patzcuarensis*.

### Descriptions of the two species

Order Calanoida

Family Diaptomidae

Subfamily Diaptominae

Genus *Mastigodiaptomus*

**Figure 5:**
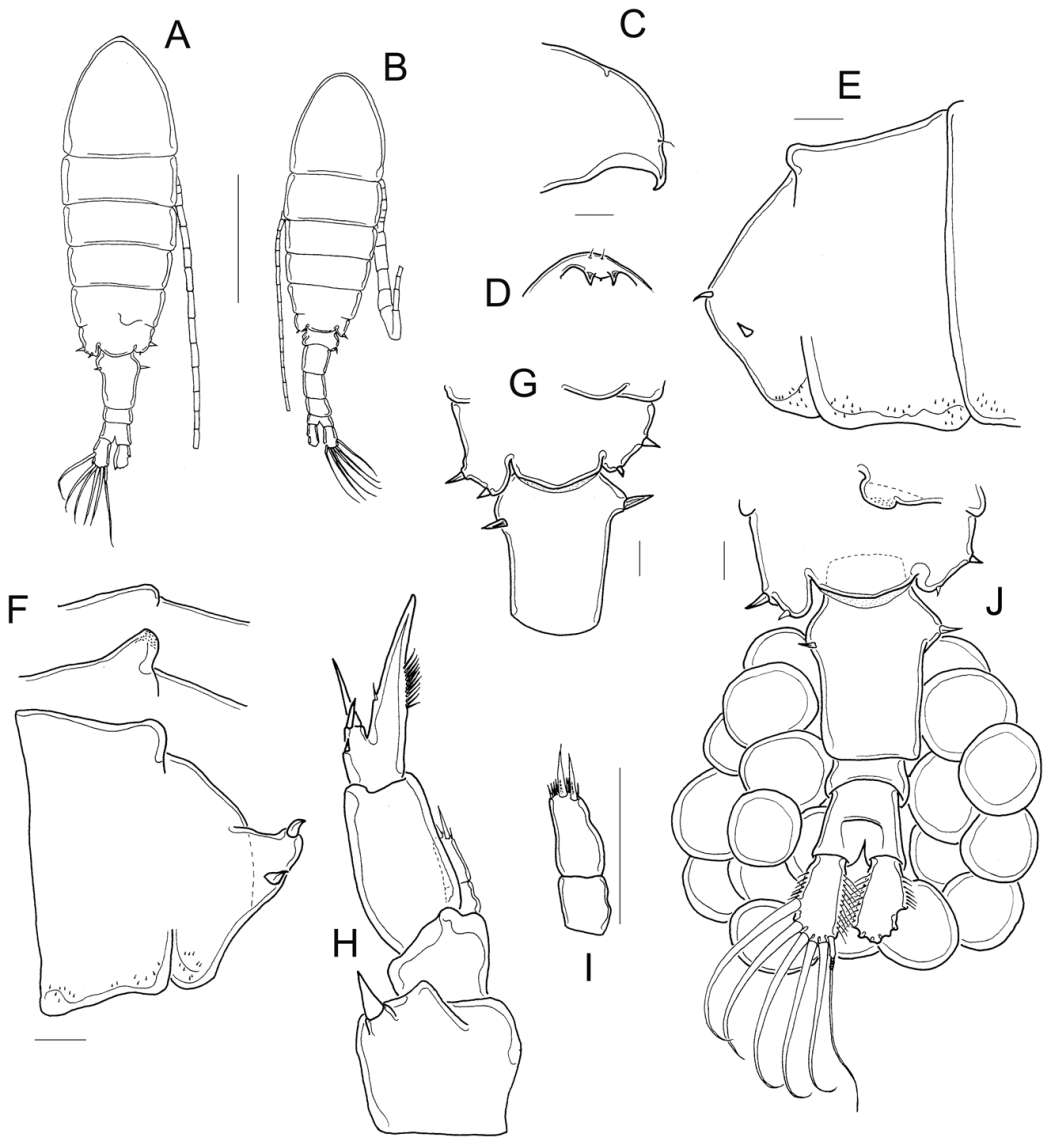
Morphology of *Mastigodiaptomus albuquerquensis* s. str. A, habitus; B, habitus; C, rostrum, lateral view; D, rostrum, frontal view; E, fourth and fifth prosomite, right; F, fourth and fifth prosomite, left; G, fifth prosomite and double-genital somite, dorsal; H, P5; I, EnpP5; J, fifth prosomite and urosome, dorsal. B: male from HTSH, Texas, A, C–G, I–J: female from HTSH, Texas; H: female from Rancho Grande, Zacatecas.

**Figure 6:**
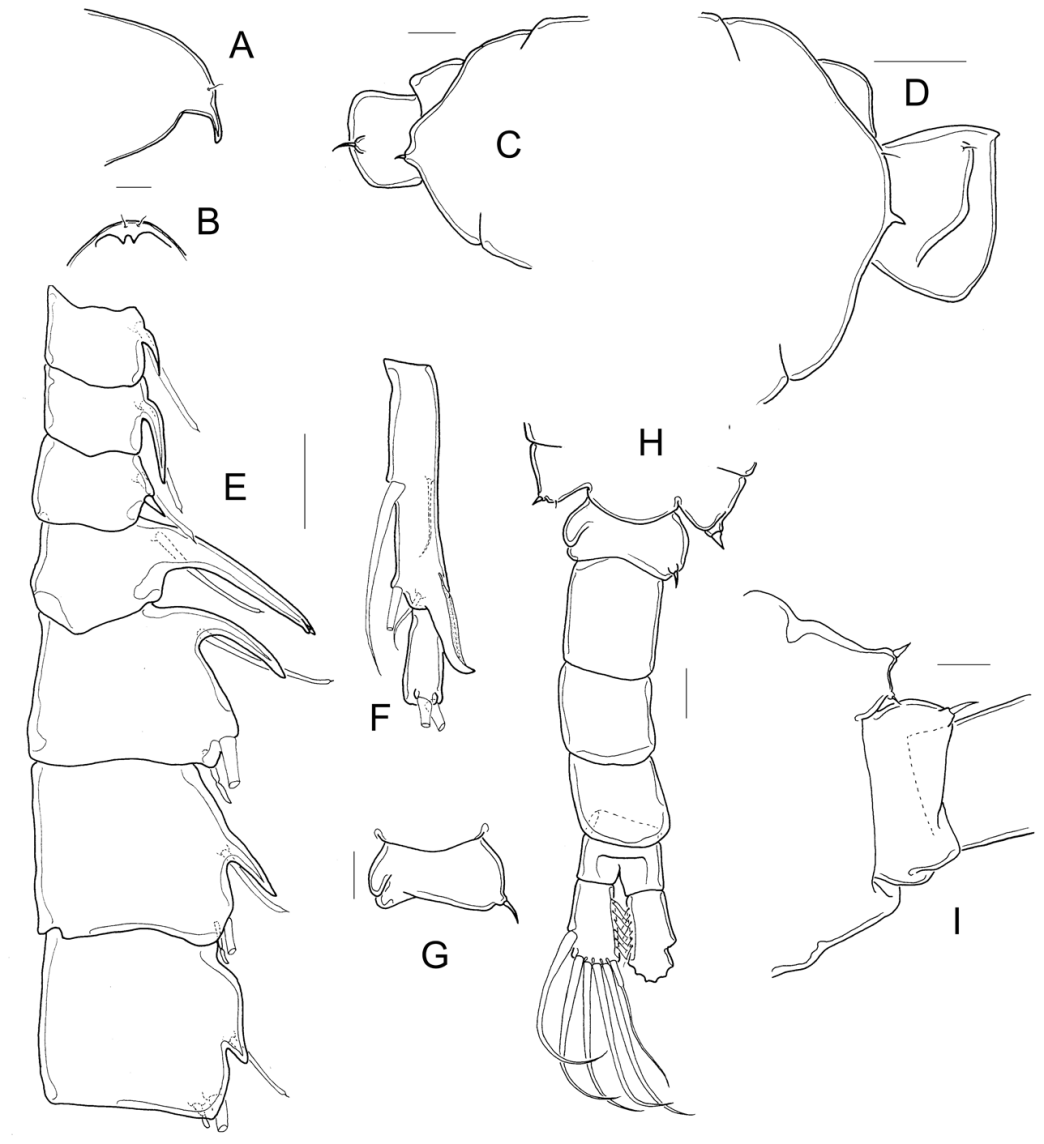
Morphology of *Mastigodiaptomus albuquerquensis* s. str. A, rostrum, lateral; B, rostrum, frontal; C, fourth and fifth prosomites and first urosomite, right; D, fourth and fifth prosomites and first urosomite, left; E, A1, segments 10–16; F, A1, segments 20–21; G, first urosomite, dorsal; H, urosomite, dorsal; I, fifth pediger, and first urosomite, dorsal. A–H: male from Rancho Grande, Zacatecas; I: male from Arizona, slide 2968 (slightly rotted to the left side).

*Mastigodiaptomus albuquerquensis* s. str. (Figs. 5, 6)

*Diaptomus albuquerquensis* Herrick, 1895

*Diaptomus* (*Mastigodiaptomus*) *albuquerquensis* Light, 1939

*Diaptomus lehmeri* Pearse, 1904

*Mastigodiaptomus albuquerquensis* (Herrick, 1895)

#### Material examined

Ten adult females and two adult males, ethanol preserved from near Rancho Grande to Zacatecas, Mexico; three adult females and five adult males from Hueco Tanks (HTSH) in Texas, USA; 12 adult females and five adult males from Laguna Bustillos, Chihuahua, Mexico; ten adult females and 4 adult males from Ignacio Ramírez dam, Estado de México; four females and three adult males from km 55 pond, Chihuahua; 14 adult females and eight adult males from El Salvador, Durango; one adult female and two adult males of the Staatliches Museum für Naturkunde Karlsruhe, from a pond near Duvey, Arizona, USA (slide numbers 2965, 2966, 2967, and 2968).

#### Females

1.47 to 1.87 mm of total body length, including Fu (Fig. 5A). Short spines on rostrum, 1.5–3.6 times longer than wide, with rounded tip (Fig. 5C, D). Antennule 25-segmented, short, ending at distal margin of anal somite (Fig. 5A); females from Zacatecas with one or two antennular segments beyond Fu (25–75 µm); in females from Ignacio Ramírez, the A1 is from the same level as the Fu to 50 µm beyond it; tiny hair-like setae on ventral surface of each prosomal somite (Fig. 5E, F).

The dorsal process of the fourth pediger is variable in shape within populations: approximately 33% of the surveyed specimens exhibit a high, frontally concave projection; 17% of females bear a low, convex projection, and the rest have a very low projection (Fig. 5F).

Distal margin of the fifth pediger delicate; central margin rounded (Fig. 5G). Right wing not projected with one dorsal and one ventral spine. Distance between the two spines is from 45 to 90 µm (Fig. 5E). Left wing is strongly projected, bearing spines separated by 32 to 75 µm (Fig. 5F).

The length ratio of Exp/Enp of P5 is 1.9 to 3.2 (average = 2.3), Enp bi-segmented, with long setae apically (Fig. 5I). Two spines on Exp2; Exp3 with spines on medial margin (Fig. 5H). Ornamentation of lateral margin of Exp3 variable: without spines (in the female from Arizona) or with one (in females from Zacatecas as in Fig. 5H) or two spines (in females from Chihuahua).

Double genital somite 1.0–15 (average = 1.2) times longer than wide; egg sacs carrying from 12 to 40 eggs. Left spine inserted dorsally on the proximal region of the somite (approximately 35% of total double genital segment length). Fu with hair-like setae on the medial margin and spine-like setae on the lateral margin (Fig. 5J).

#### Males

1.37–1.82 mm total body length, including Fu; cephalothorax length represents 69.5–70% of total body length. Left A1 short, not reaching the furcal rami (Fig. 5B). Short spines on rostrum, 2.6 times as long as wide (Fig. 6A, B). Cuticular surface smooth, no tiny spines on prosomal somites (Fig. 6C, D).

Right A1 22-segmented, with spiniform process on segments 10, 11, 13, 14, 15, and 16 (Fig. 6E). Spine on segment 10 short, does not reach distal margin of the bearing segment. Segment 20 bearing a hook-like projection with a smooth hyaline membrane; L/W ratio of segment 20 is 3.2 to 5.0 (Fig. 6F).

First urosomal segment with one strong, curved spine on right side, averaging 4.6 times longer than wide (Fig. 6G); left side bearing one tiny, hair-like seta only observable laterally. A dorsal, thin spine present on right wing in males from Arizona, Laguna Bustillos, and Hueco Tanks (Fig. 6I); absent in males from Zacatecas (Fig. 6H). Left wing with one strong, ventral spine plus one weak, hair-like seta dorsally (Fig. 6H).

Fourth urosomite projected, but this projection not strongly bulbose. Fu pilose on medial margin (Fig. 6H).

**Figure 7:**
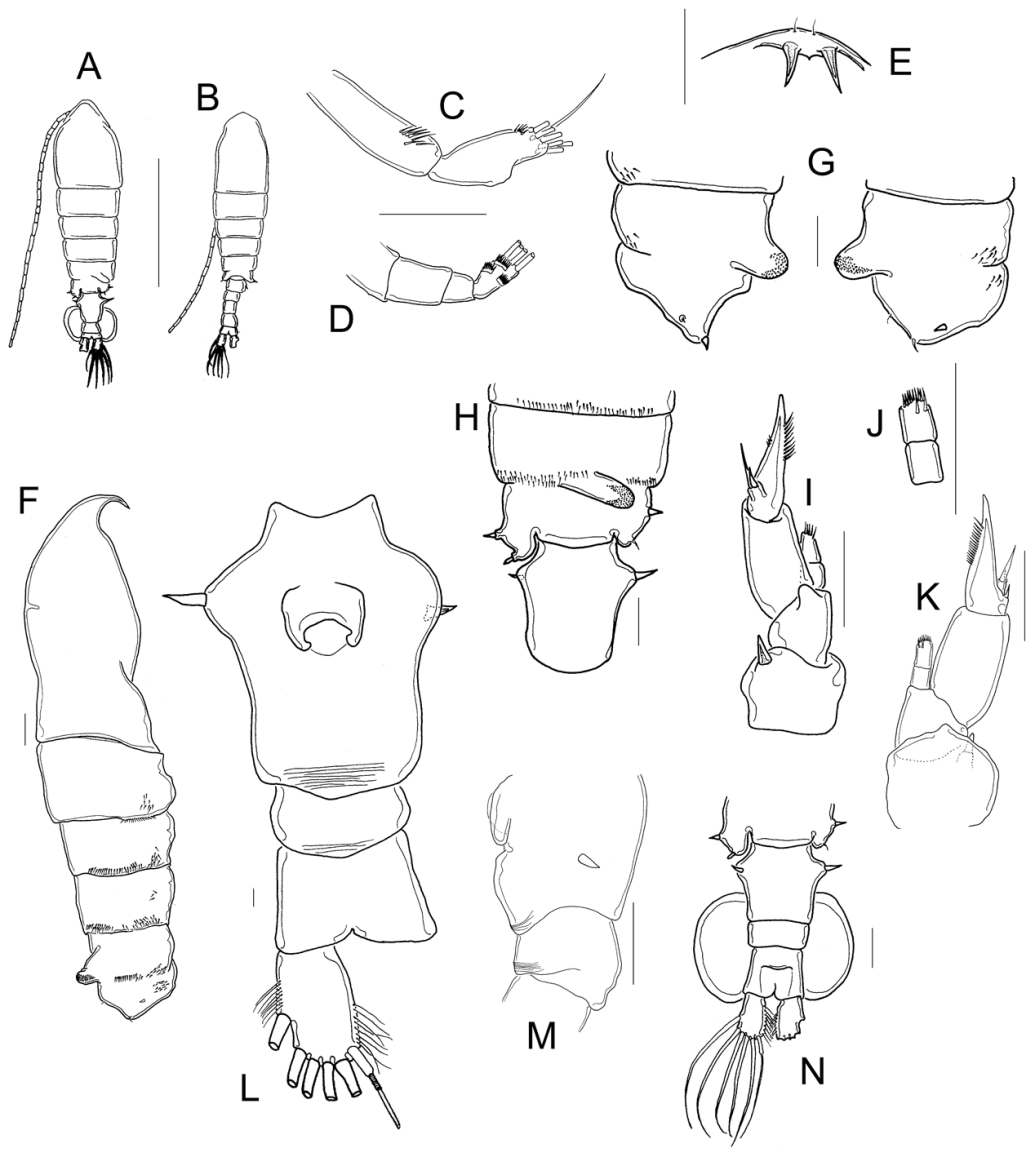
Morphology of *Mastigodiaptomus patzcuarensis*. A, habitus; B. habitus; C, EnpA2; D, detail of Enp2 of mandible; E, rostrum, frontal; F, prosome, lateral; G, fourth and fifth prosomites, left and right sides; H, fourth and fifth prosomites and genital double-somite, dorsal; I, P5; J, EnpP5; K, P5; L, urosome, ventral; M, urosome, lateral; N, urosome, dorsal. A, E–J, N: females from Pátzcuaro; B: male from Pátzcuaro; C, D, K, M: typus of *M. albuquerquensis patzcuarensis*, slide 4058.

**Figure 8:**
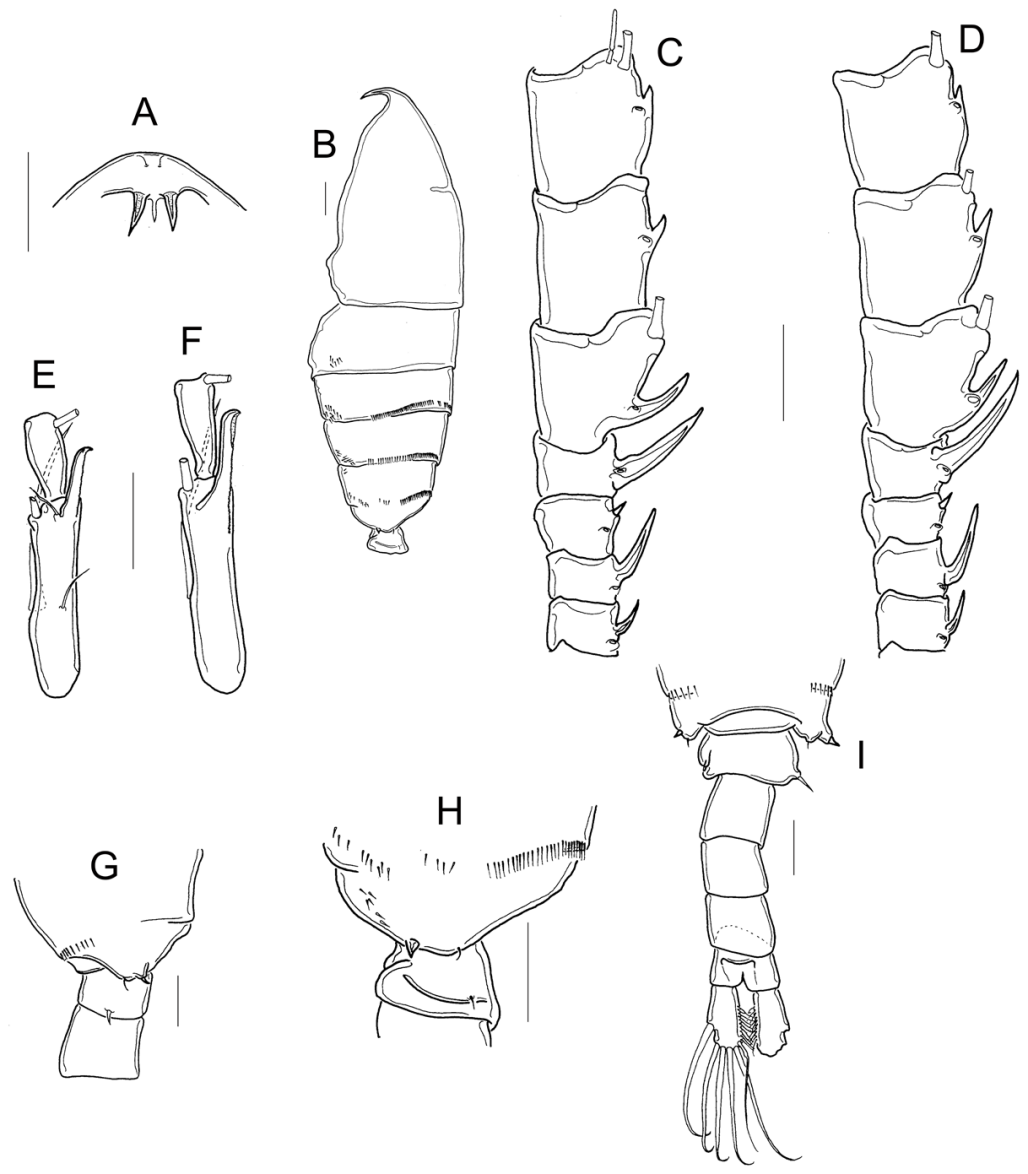
Morphology of *Mastigodiaptomus patzcuarensis*. A, rostrum, frontal; B, prosome, lateral; C, D, A1, segments 10–16; E, F, A1, segments 20–21; G, fourth pediger and first urosomite, right; H, fourth pediger and first urosomite, left; I, urosomite, dorsal. A–C, E, G–H: male from Pátzcuaro; D, F: male from Cuitzeo.

*Mastigodiaptomus patzcuarensis* (Kiefer, 1938), **stat. n.** urn:lsid:zoobank.org:act:0AE248EE-16FB-4359-92B9-6FB3DD7477F7 (Figs. 7, 8).

Syn: *Diaptomus albuquerquensis patzcuarensis* Kiefer, 1938.

#### Material examined

15 adult females and ten adult males, ethanol preserved from Pátzcuaro, Michoacán; 15 adult females and 11 adult males from Cuitzeo, Michoacán; nine adult females and five adult males from La Cruz, Guanajuato; two adult females and six adult males from La Goleta, Estado de Mexico; three adult females and two adult males of the Staatliches Museum für Naturkunde Karlsruhe, from Pátzcuaro, Michoacán, México (slides labeled as “*Mastigodiaptomus albuquerquensis patzcuarensis*, Typus” by Kiefer number 4057 – one dissected female, 4058 – two adult females, 4059 – one dissected male, and 4060 –one adult male).

#### Females

Total body length is 0.9–1.3 mm (average = 1.14 mm), including Fu (Fig. 7A). In the type material, it was possible to observe a group of spine-like setae on Enp1 in the antennae (Fig. 7C) and the three toothed pectens on the outer margin of mandibular Enp2 (Fig. 7D).

Rostrum with spines 3.6 times longer than wide (Fig. 7E). A1 long: one to three antennular segments (25 to 175 µm) are beyond the distal margin of Fu (Fig. 7A). Cuticle surface with hair-like setae on ventral and distal margins from second to fifth prosomal somites in females from Pátzcuaro (Fig. 7F) but nude in females from La Cruz, Cuitzeo, and Goleta. Dorsal process of fourth prosomal somite always convex anteriorly, pitted (Fig. 7G).

Central margin of fifth pediger slightly angled (Fig. 7H). Right wing with two spines. Distance between spines of right wing 21.5–52.5 µm (Fig. 7G right). Left wing projected, spines on left wing separated by 17–40 µm (Fig. 7G left).

Exp1 of P5 is 1.7–2.1 times longer than the bi-segmented Enp (average = 1.8) (Fig. 7J). The Enp is 4–7 times longer than the very short apical setae; Exp3 with spines on medial margin and a reduced group of spines (Fig. 7I) or no spines on the lateral margin (Fig. 7K). Genital double somite with parallel scars ventrally (Fig. 7L, M), 1.2 times longer than wide, carrying 2–4 eggs in the sac (Fig. 7N). Left spine inserted dorsal or ventrally in a high position on the genital double somite (approximately 40% of total length segment). Hair-like setae along the lateral and medial margins of the furcal rami; these setae of similar length in both margins (Fig. 7L).

#### Males

Total body length is 0.92–1.1 mm; left A1 short, reaching the tip of furcal rami, or shorter; the prosome represents approximately 70% of total body length (Fig. 7B). Spines on rostrum 3.5–5.0 times longer than wide (Fig. 8A). Cuticle surface as described for females, with hair-like seta in males from Pátzcuaro (Fig. 8B), but bare in males from La Cruz, Goleta, and Cuitzeo. Right A1 with spines on segments 10, 11, and 13–16. Spine on segment 10 of A1, trespassing distal margin of segment (Fig. 8C, D). Length ratio of spines on segments 10/11 is 0.5. Segment 20 bearing a hook-like projection with a thin, serrated hyaline membrane; L/W ratio of 20^th^ segment is 3.3–4.8 (4.3 on average) (Fig. 8E, F).

Right wing of fifth pediger with two spines: one well developed ventral spine and one tiny dorsal spine (Fig. 8G). Left wing with similar ornamentation (Fig. 8H). First abdominal segment with one strong or short spine on the right side (Fig. 8I, G). Left side with one tiny seta (Fig. 8H). Right, dorsal projection of fourth abdominal segment not strongly bulbose. Medial margin of Fu pilose (Fig. 8I).

## Discussion

The detailed analyses of the morphology described above (and supplemented in Tables 3 and 4) show slight but important differences between the two species, in agreement with the barcoding.

**Table 3:** Morphometric characters in the surveyed females.

Feature/Population	*M. albuquerquensis* s. str.	*M. patzcuarensis*
Total body length	1700 (1475–1875 µm)	1141 (975–1375 µm)
Rostrum, length/width ratio of spines	2.51 (1.5–3.6)	3.61 (2.6–4.0)
Genital segment; length/width ratio	1.2 (1.0–1.5)	1.2 (1.1–1.3)
Furcal rami length	111.5 (97.5–115 µm)	65.7 (55–77.5 µm)
Length ratio: EnpP5/apical setae of EnpP5	3.7 (2.5–5.6)	5.8 (4–7)
Position of left spine in comparison with genital double-segment length	35.5 (25–65%)	39.5 (30.3–49.2%)
Position of right spine in comparison with genital double-segment length	29.4 (20–40%)	32.8 (29.3–38.0%)
Distance between spines in right wing	68.9 (45–90 µm)	40.5 (21.5–52.5 µm)
Distance between spines in left wing	47.6 (32.5–75 µm)	27.9 (17.5–40 µm)
Number of eggs carried in ovisac	23.9 (12–40)	3.1 (2–4)

Average and minimum-maximum (within brackets) values are presented.

**Table 4:** Morphometric characters in the surveyed males.

Feature/Population	*M. albuquerquensis* s. str.	*M. patzcuarensis*
Total body length	1594 (1375–1825 µm)	1042 (925–1159 µm)
Furcal length	525 (450–600 µm)	324 (275–375 µm)
Rostrum, length/width ratio of spines	2.6 (1.74–4.0)	4.1 (3.5–5.0)
A1 length ratio spines on segment 10/11	0.56 (0.45–0.66)	0.57 (0.5–0.6)
A1, segment 21 length/segment 20 projection length	1.4 (1.12–1.78)	1.4 (1.0–1.72)
Right A1, segment 20 length/width ratio	4.1 (3.26–5.0)	4.4 (3.3–4.8)
Length/width ratio of right spine of first urosomite	4.6 (3.3–5.5)	3.9 (2–5.0)

Average and minimum-maximum (within brackets) values are presented.

However, the barcoding results suggest the presence of one more species from Central Mexico, herein named *M.* cf. *albuquerquensis*, with disjunctive populations from the other two. Although the specimens were analyzed in detail by their morphological attributes, it was not possible to ascertain whether they belong to a different species. It is possible these specimens correspond to another species described as *Mastigodiaptomus lehmeri* Pearse, 1904 [8], and later synonymized with *M. albuquerquensis* [21]. Unfortunately, type material for *M. lehmeri* is nonexistent, and the type locality is quite vague, stated simply as Mexico City. All systems with *M.* cf. *albuquerquensis* in this study are from localities less than 150 km from Mexico City, except for the specimens from Cuitzeo. To disentangle this latter species, it is necessary to prepare hybridization assays among the three species similar to those previously prepared to uncover *Leptodiaptomus garciai*, an endemic from a saline crater lake [10]. If this result indicates a different biological species, it would be the first *Mastigodiaptomus* with no morphological features allowing identification to species level. This analysis will be part of a future work due to the need to work with live specimens collected as close as possible to Mexico City.

On the other hand, the micro-structural conformation of anatomic features related to reproduction (i.e., antennules and fifth legs) in female and male freshwater copepods has been informative for species recognition elsewhere [20], [22], and these morphological differences are consistent with reproductive isolation [10] or genetic differentiation in the COI gene among species [23]. Both genetic analyses and morphology, have been employed to successfully discriminate sibling species of marine and freshwater copepods [24], [25], cladocerans [23], rotifers [26], polychaetes [27], and others. These descriptions based on morphology and molecular data, are part of the integrative taxonomy [28], a new approach where different types of evidence converge to support the species discovery that currently is revitalizing this discipline [29].

### Morphological features

Following the description of *Mastigodiaptomus albuquerquensis* [11], the hyaline membrane inserted on the caudal side of right basipodite of the male fifth leg (which is butterfly-like) was widely used to identify to this species regionally [1]. However, the genetic analysis in the present survey has shown that at least two different species share the same morphological structure in the fifth leg in males, in the genital field in females, and in all the mouthparts and the antennular ornamentation in both sexes. Therefore, additional features must be considered to distinguish species; for instance, the analysis of tegument features has been suggested to distinguish *Mastigodiaptomus* species [30]. According to this idea, and following the information obtained with the gen COI and PCA analyses, the combination of the following morphological characteristics is present in *M. albuquerquensis* s. str.: females with hair-like setae on the lateral margin of the Fu shorter than the setae on the medial margin; prosomites with very short hair-like setae on the ventral region; short rostral spines; A1 ending before the distal-most margin of Fu, central margin of fifth prosomite rounded, delicate; dorsal process of fourth pediger variable in shape within the populations; fifth leg endopod bearing a long distal seta; and ovisac carrying a large number of eggs (12–40). In addition to the tegumental features present in females, males of *M. albuquerquensis* have a smooth hyaline membrane on the 20^th^ right antennular segment, and the spine of the 10^th^ segment is short, not reaching the distal margin of the bearing segment.

In contrast, females of *M. patzcuarensis* can be distinguished from those of *M. albuquerquensis* s. str. by the following combination of characters: the hair-like setae on lateral and medial Fu margins with the same length; prosomites with long, hair-like setae along the distal margin; long rostral spines; A1 ending after the distal-most margin of Fu, central margin of fifth prosomite angled; shape of the dorsal process of the fourth pediger always the same within populations (high with concave anterior margin); fifth leg endopod bearing a short distal seta; and ovisac carrying a small number of eggs (2–4). Males of *M. albuquerquensis* have a thin, serrated hyaline membrane on the 20^th^ right antennular segment, and the spine tip of the 10^th^ segment is beyond the distal margin of the bearing segment.

For freshwater copepods, the differences in body length have been considered to be due to the limitation of nutrients [31], trophic state [32], or predation [32], [33]. However, in populations inhabiting tropical latitudes, this variation does not exceed dozens of microns within the same population (across seasonal changes) or between differentially distributed populations. When *M. albuquerquensis* is compared with *M. patzcuarensis*, the body length varies by at least 600 µm; a corporal difference of this magnitude can be used as an additional feature to distinguish between these species.

### Molecular support contrasted with morphology

For the molecular Id tree, the aligned matrix consisted of 615 characters, of which 171 (27.8%) were variable and 86 (14%) were parsimony informative. *Mastigodiaptomus albuquerquensis* forms a consistent group, and the two subgroups below it are the specimens from Papasquiaro B and Rancho Grande to Zacatecas—are also regarded as *M. albuquerquensis*. Morphologically, the specimens from Zacatecas exhibited three variations compared with the rest of populations from northern Mexico: A1 was longer in females, some females had two spines (not one) on the left side of Enp3P4, and the right wing of the last prosomite in the surveyed males had one element (not two). These variations might be at the specific level, but currently, it is not possible to affirm that they will lead to reproductive isolation.

A similar phenomenon may be occurring in the other major cluster divided in two: one represented by *M. patzcuarensis* and the other by *M.* cf. *albuquerquensis*. The lack of long hair-like setae on the prosomites in females and males from Goleta, La Cruz, and Cuitzeo are most likely indicative of a different species, but it is first necessary to diagnose *M. lehmeri* from Mexico City.

In addition, to draw definite identity about the two clusters from the north, it is necessary to conduct an experimental design to establish whether biological isolation exists between them, as suggested by the barcodes. Such an experiment is a formidable task in semi-desert regions where no previous methods have been developed to hatch the diapause stages of this type of copepods and where the water in pools sometimes requires several years to appear, depending on the rainfall regime.

In conclusion, other possible species within the *albuquerquensis* complex have yet to be clarified. In spite of this problem, we consider that these results will contribute to our understanding of the limited distribution of the freshwater zooplankton, in this case a calanoid, previously regarded as a widespread species in the Americas.

## Supporting Information

Figure S1 **BOLD TaxonID Tree.**
(PDF)

Table S1 Localities and sequence access for M. albuquerquensis s. l., collected in Mexico (*some previously published [5]). NS: Data available only in BOLD database.
(DOC)
