# Supplementary material for: An Example of How Barcodes Can Clarify Cryptic Species: The Case of the Calanoid Copepod Mastigodiaptomus albuquerquensis (Herrick)
Source: PLoS One. 2014 Jan 21;9(1):e85019. doi: 10.1371/journal.pone.0085019 (PMC3897401; doi:10.1371/journal.pone.0085019)

BOLD TaxonID Tree

Title	:	SEARCH: Process ids(53 ids) [SEARCH2]
Date	:	17-September-2013
Data Type	:	Nucleotide
Distance Model	:	Kimura 2 Parameter
Marker	:	COI-5P
Codon Positions	:	1st, 2nd, 3rd
Labels	:	SampleID, ProcessID, Sequence Length, BIN guid
Filters	:	Length > 200
Colorization	:	[blue]=Stop Codons [red]=Contamination or misidentification

Sequence Count	:	53
Species count	:	3
Genus count	:	1
Family count	:	1
Unidentified	:	0

BIN Count	:	5

SEARCH:_Process_ids(53_ids)_[SEARCH2] Tue Sep 17 17:07:20 2013 Page 1 of 1

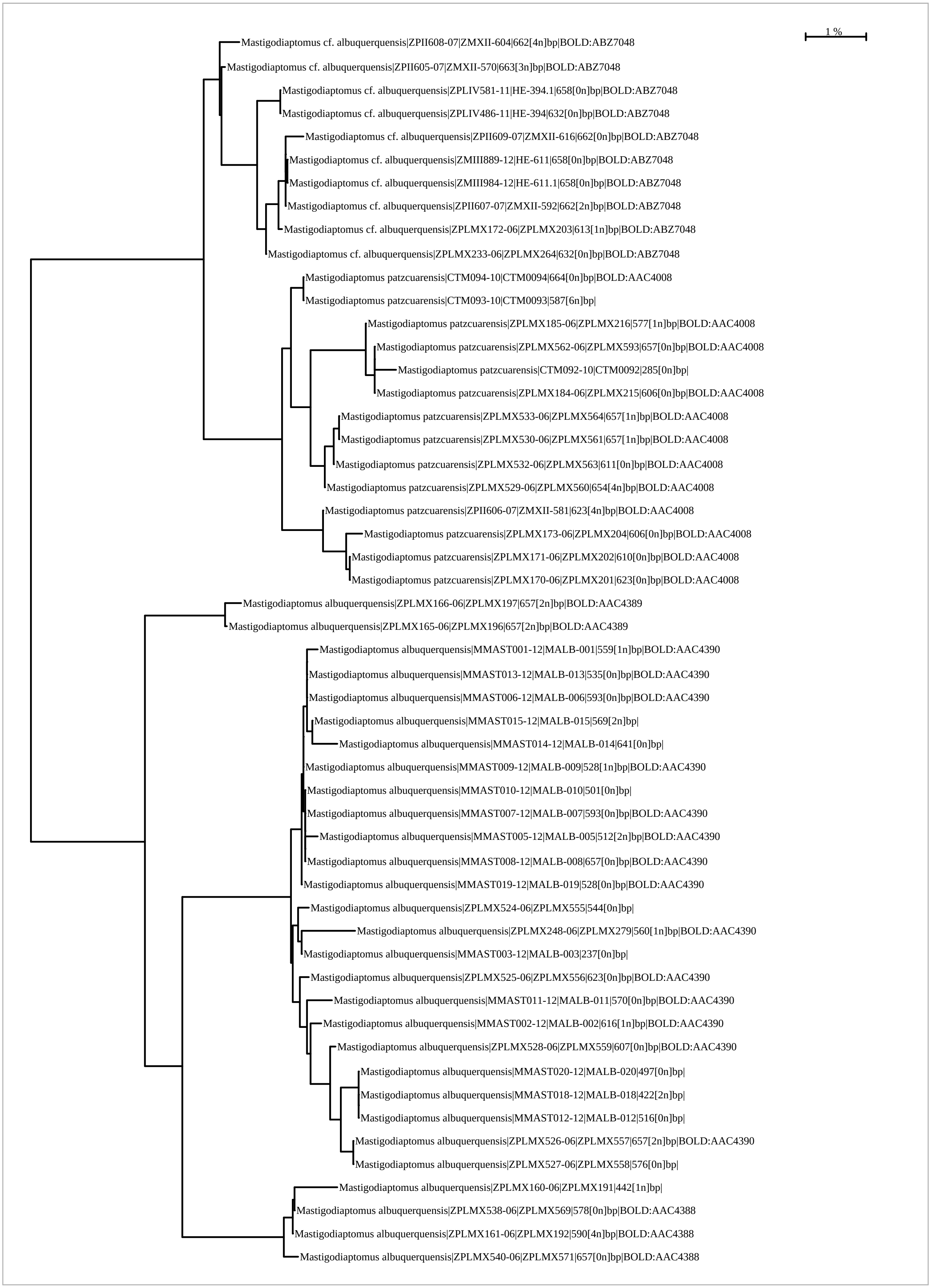

Supplement: Figure S1 — BOLD TaxonID Tree. (PDF) [file pone.0085019.s001.pdf]
