# Supplementary material for: An Example of How Barcodes Can Clarify Cryptic Species: The Case of the Calanoid Copepod Mastigodiaptomus albuquerquensis (Herrick)
Source: PLoS One. 2014 Jan 21;9(1):e85019. doi: 10.1371/journal.pone.0085019 (PMC3897401; doi:10.1371/journal.pone.0085019)
Supplement: Table S1 — Localities and sequence access for M. albuquerquensis s. l., collected in Mexico (*some previously published [5]). NS: Data available only in BOLD database. (DOC) [file pone.0085019.s002.doc]

Table_S1.- Localities and sequence access for *M. albuquerquensis* s. l., collected in Mexico (*some previously published [5]). NS: Data available only in BOLD database.

| Species | Process Id BOLD | GenBank Access Number | Locality | Lat N | Long W |
| --- | --- | --- | --- | --- | --- |
| *Mastigodiaptomus* *patzcuarensis* | CTM092-10 | NS | Pátzcuaro Lake, central | 19.60 | 101.65 |
| *Mastigodiaptomus* *patzcuarensis* | CTM093-10 | NS | Pátzcuaro Lake, central | 19.60 | 101.65 |
| *Mastigodiaptomus* *patzcuarensis* | CTM094-10 | HQ944364 | Pátzcuaro Lake, central | 19.60 | 101.65 |
| *Mastigodiaptomus* *albuquerquensis* | MMAST001-12 | KC860774 | Km 55 Ciudad Juarez to Casas Grandes | 31.55 | 107.18 |
| *Mastigodiaptomus* *albuquerquensis* | MMAST002-12 | KC860773 | Km 55 Ciudad Juarez to Casas Grandes | 31.55 | 107.18 |
| *Mastigodiaptomus* *albuquerquensis* | MMAST003-12 | NS | Km 55 Ciudad Juarez to Casas Grandes | 31.55 | 107.18 |
| *Mastigodiaptomus* *albuquerquensis* | MMAST005-12 | KC860772 | Km 55 Ciudad Juarez to Casas Grandes | 31.55 | 107.18 |
| *Mastigodiaptomus* *albuquerquensis* | MMAST006-12 | KC860771 | Km 55 Ciudad Juarez to Casas Grandes | 31.55 | 107.18 |
| *Mastigodiaptomus* *albuquerquensis* | MMAST007-12 | KC860770 | Km 55 Ciudad Juarez to Casas Grandes | 31.55 | 107.18 |
| *Mastigodiaptomus* *albuquerquensis* | MMAST008-12 | KC860769 | Km 55 Ciudad Juarez to Casas Grandes | 31.55 | 107.18 |
| *Mastigodiaptomus* *albuquerquensis* | MMAST009-12 | KC860768 | Km 55 Ciudad Juarez to Casas Grandes | 31.55 | 107.18 |
| *Mastigodiaptomus* *albuquerquensis* | MMAST010-12 | KC860767 | Km 55 Ciudad Juarez to Casas Grandes | 31.55 | 107.18 |
| *Mastigodiaptomus* *albuquerquensis* | MMAST011-12 | KC860766 | Laguna Bustillos | 28.56 | 106.71 |
| *Mastigodiaptomus* *albuquerquensis* | MMAST012-12 | KC860765 | Laguna Bustillos | 28.56 | 106.71 |
| *Mastigodiaptomus* *albuquerquensis* | MMAST013-12 | KC860764 | Laguna Bustillos | 28.56 | 106.71 |
| *Mastigodiaptomus* *albuquerquensis* | MMAST014-12 | KC860763 | Laguna Bustillos | 28.56 | 106.71 |
| *Mastigodiaptomus* *albuquerquensis* | MMAST015-12 | NS | Laguna Bustillos | 28.56 | 106.71 |
| *Mastigodiaptomus* *albuquerquensis* | MMAST018-12 | NS | Laguna Bustillos | 28.56 | 106.71 |
| *Mastigodiaptomus* *albuquerquensis* | MMAST019-12 | KC860762 | Laguna Bustillos | 28.56 | 106.71 |
| *Mastigodiaptomus* *albuquerquensis* | MMAST020-12 | NS | Laguna Bustillos | 28.56 | 106.71 |
| *Mastigodiaptomus* cf. *albuquerquensis* | ZMIII889-12 | NS | Ignacio Ramirez Dam, littoral | 19.46 | 99.78 |
| *Mastigodiaptomus* cf. *albuquerquensis* | ZMIII984-12 | NS | Ignacio Ramirez Dam, littoral | 19.46 | 99.78 |
| *Mastigodiaptomus* cf. *albuquerquensis* | ZPII605-07 | NS | Flor del Bosque | 19.04 | 98.23 |
| *Mastigodiaptomus* *patzcuarensis* | ZPII606-07 | NS | Flor del Bosque | 19.04 | 98.23 |
| *Mastigodiaptomus* cf. *albuquerquensis* | ZPII607-07 | NS | Flor del Bosque | 19.04 | 98.23 |
| *Mastigodiaptomus* cf. *albuquerquensis* | ZPII608-07 | NS | Flor del Bosque | 19.04 | 98.23 |
| *Mastigodiaptomus* cf. *albuquerquensis* | ZPII609-07 | NS | Flor del Bosque | 19.04 | 98.23 |
| *Mastigodiaptomus* cf. *albuquerquensis* | ZPLIV486-11 | KC617679 | Ignacio Ramirez Dam | 19.46 | 99.80 |
| *Mastigodiaptomus* cf. *albuquerquensis* | ZPLIV581-11 | KC617680 | Ignacio Ramirez Dam | 19.46 | 99.80 |
| *Mastigodiaptomus* *albuquerquensis* | ZPLMX160-06* | EU770479 | Rancho Grande to Zacatecas | 23.22 | 102.87 |
| *Mastigodiaptomus* *albuquerquensis* | ZPLMX161-06* | EU770480 | Rancho Grande to Zacatecas | 23.22 | 102.87 |
| *Mastigodiaptomus* *albuquerquensis* | ZPLMX165-06* | EU770484 | Papasquiaro B | 24.51 | 104.66 |
| *Mastigodiaptomus* *albuquerquensis* | ZPLMX166-06* | EU770485 | Papasquiaro B | 24.51 | 104.66 |
| *Mastigodiaptomus* *patzcuarensis* | ZPLMX170-06* | EU770491 | Cuitzeo | 19.95 | 101.13 |
| *Mastigodiaptomus* *patzcuarensis* | ZPLMX171-06* | EU770492 | Cuitzeo | 19.95 | 101.13 |
| *Mastigodiaptomus* cf. *albuquerquensis* | ZPLMX172-06* | EU770493 | Cuitzeo | 19.95 | 101.13 |
| *Mastigodiaptomus* *patzcuarensis* | ZPLMX173-06* | EU770494 | Cuitzeo | 19.95 | 101.13 |
| *Mastigodiaptomus* *patzcuarensis* | ZPLMX184-06* | EU770495 | La Goleta | 20.07 | 99.56 |
| *Mastigodiaptomus* *patzcuarensis* | ZPLMX185-06* | EU770496 | La Goleta | 20.07 | 99.56 |
| *Mastigodiaptomus* cf. *albuquerquensis* | ZPLMX233-06* | EU770497 | Cuitzeo | 19.92 | 101.14 |
| *Mastigodiaptomus* *albuquerquensis* | ZPLMX248-06* | EU770486 | El Salvador Dgo limnetic | 26.07 | 104.97 |
| *Mastigodiaptomus* *albuquerquensis* | ZPLMX524-06 | NS | El Salvador | 26.07 | 104.97 |
| *Mastigodiaptomus* *albuquerquensis* | ZPLMX525-06* | EU770477 | El Salvador | 26.07 | 104.97 |
| *Mastigodiaptomus* *albuquerquensis* | ZPLMX526-06* | EU770481 | El Salvador Dgo limnetic | 26.07 | 104.97 |
| *Mastigodiaptomus* *albuquerquensis* | ZPLMX527-06 | NS | El Salvador | 26.07 | 104.97 |
| *Mastigodiaptomus* *albuquerquensis* | ZPLMX528-06* | EU770478 | El Salvador | 26.07 | 104.97 |
| *Mastigodiaptomus* *patzcuarensis* | ZPLMX529-06* | EU770488 | La Cruz I Gto | 21.19 | 100.57 |
| *Mastigodiaptomus* *patzcuarensis* | ZPLMX530-06* | EU770489 | La Cruz I Gto | 21.19 | 100.57 |
| *Mastigodiaptomus* *patzcuarensis* | ZPLMX532-06* | EU770487 | La Cruz I | 21.19 | 100.57 |
| *Mastigodiaptomus* *patzcuarensis* | ZPLMX533-06* | EU770490 | La Cruz I Gto | 21.19 | 100.57 |
| *Mastigodiaptomus* *albuquerquensis* | ZPLMX538-06* | EU770482 | Rancho Grande to Zacatecas | 23.22 | 102.87 |
| *Mastigodiaptomus* *albuquerquensis* | ZPLMX540-06* | EU770483 | Rancho Grande to Zacatecas | 23.22 | 102.87 |
| *Mastigodiaptomus* *patzcuarensis* | ZPLMX562-06* | EU770498 | La Goleta | 20.07 | 99.56 |
